# Comprehensive Analysis of Role of Cyclin-Dependent Kinases Family Members in Colorectal Cancer

**DOI:** 10.3389/fonc.2022.921710

**Published:** 2022-06-22

**Authors:** Liping Guan, Yuanyuan Tang, Guanghua Li, Zhao Qin, Shaoshan Li

**Affiliations:** ^1^School of Basic Medical Sciences, Xinxiang Medical University, Xinxiang, China; ^2^Henan International Joint Laboratory of Immunity and Targeted Therapy for Liver-Intestinal Tumors, Xinxiang Medical University, Xinxiang, China; ^3^Department of General Surgery of the Third Affiliated Hospital of Xinxiang Medical University, Xinxiang Medical University, Xinxiang, China

**Keywords:** colorectal cancer, CDK, biomarkers, infiltration of immune cells, therapeutic targets

## Abstract

**Background:**

Cyclin-dependent kinases (CDKs) are cell cycle regulators, and abnormal activation can accelerate tumor cell proliferation. However, The relation between CDKs dysregulation to colorectal cancer incidence and progression have not been examined in detail. Methods:Differences in CDKs expression between colorectal cancer and normal tissues, associations between expression and clinical prognosis, incidence and frequencies of CDKs gene mutations, and the influences of CDKs on tumor infiltration by immune cells were examined by analyses of Oncomine, Gene Expression Profiling Interactive Analysis, Kaplan-Meier plotter, cBioPortal, GeneMANIA, and TIMER databases.

**Results:**

Colorectal cancer tissues showed enhanced expression levels of CDKs 1/2/4/5/6/8/12/13/19 but reduced CDK3 expression. CDK7 was highly expressed in some colorectal cancer tissues but downregulated in others. Expression levels of CDK1/3/4/7/8/10/11b/13/18/19/20 were correlated with clinical stage, and CDK 5/10/12/16 expression levels predicted prognosis and survival. Differential CDKs expression correlated with cell cycle progression, amino acid polypeptide modifications, and activation of other protein kinases. Expression levels of all CDKs except CDK16 were correlated with infiltration of CD4+T, CD8+T, B and Tregs cells.

**Conclusions:**

CDK 1 and 4 could be used as diagnostic biomarkers for CRC. CDK 5/10/12/16 can be utilized as prognostic biomarkers.

## Background

Abnormal cell cycle regulation is one of the major causes of tumor cell hyper-proliferation and tumor growth. Cyclin-dependent kinases (CDKs) are critical cell cycle regulators and are overexpressed in a variety of tumor types, strongly suggesting major contributions to cell cycle acceleration and ensuing tumor initiation and (or) progression (1, 2). Indeed, targeting CDKs has been shown to restore normal cell cycle progression and inhibit tumor cell growth. For instance, several selective small molecule inhibitors of CDK 4 and 6 (Palbociclib, Ribociclib, and Abemaciclib) have been approved by the United States Food and Drug Administration (FDA) for the treatment of advanced or metastatic breast cancer (3–5). At present, additional small molecule inhibitors of CDKs are being tested in preclinical or clinical trials for the treatment of lung cancer, prostate cancer, and other neoplastic diseases (6, 7). Compared to other types of cancers, however, pathogenic functions of CDKs in colorectal cancer (CRC) have not been extensively investigated.

In recent years, more than hundreds of biomarkers have been found for CRC, including mast cells (MCs), DNA, RNA, microRNA, epigenetic changes and protein etc (8, 9). However, few biomarkers really benefits patients. Mutant KRAS, TP53, APC, and microsatellite instability markers (MSI) might contribute to clinical therapy, less to early diagnosis. MicroRNA, derived from blood and feces, is considered to be an early and non-invasive marker for CRC, but low specificity limit its application, and the combination multiple microRNA can slightly improve its specificity (10). Hence,the high specificity of CRC markers still need to be developed. In this study, CDKs expression profiles and the clinical impact of CDKs dysregulation on colorectal cancer prognosis were comprehensively analyzed to provide novel biomarker for diagnosis and treatment.

## Methods and Materials

### Oncomine (www.oncomine.org)

To analyze the mRNA levels of CDKs in diverse cancer types, an open access database Oncomine (11) was used. Oncomine is well-developed tumor expression spectroscopic database, with a total of microarray data collected for 715 cancers, involving 19 different cancer types. In this study, 237 samples were included in the study. Threshold of data mining ①tumor type: colorectal cancer, ②specimen type: surgical resection specimens, ③tissue comparison: colorectal cancer vs normal breast tissue, ④data type: mRNA, ⑤significance: P<0.05, ⑥differential expression level: greater than 2-fold, ⑦gene row Order: Top 10%. T-test was used to compare the expression of CDKs in CRC and normal tissue.

### GEPIA (http://gepia.cancer-pku.cn/)

GEPIA (Gene Expression Profiling Interactive Analysis) is a gene expression profiling data dynamic analysis database, including more than 9000 tumor samples (275 are the CRC samples)and more than 8,000 normal samples from TCGA and GTEx. In this work, 275 CRC samples and 375 normal samples were used to analyze the differential gene expression of CDKs between tumor and normal tissues, pathological stage analysis, correlative prognostic and patient survival analysis in CRC patients (12). Student’s t-test was used to analyze the differential expression in the expression of CDKs in CRC, and Spearman was used for correlation between pathological stage analysis and CDKs.

### Kaplan-Meier plotter (http://kmplot.com/private/)

In this work, overall survival rate(OS) and disease free survival (DFS) of 165 CRC clinical cases were analyzed using Kaplan-Meier plotter with 50% cutoff for both low and high-expression groups. Kaplan-Meier model was used to analyze the relationship between CDKs expression and patient prognosis, and the Log rank test was used to evaluate whether the survival distribution varied between groups if the precondition of equiproportional risk was met, otherwise, the Land mark test was used.

### CBioPortal (https://www.cbioportal.org/)

cBioPortal is an open-access resource with data derived from multiple data platforms, including somatic mutations, DNA copy number alteration, mRNA and DNA methylation (13). In this work, we got a total of 1949 samples from three CRC datasets in cBioPortal, and then gene alterations of CDKs were analyzed in CRC samples.

### GeneMANIA (http://www.genemania.org)

Gene MANIA website (14) was used to analyse the function of CDKs, and evaluate the predictive value of CDKs. Genes with possible interactions with CDKs were acquired through the GeneMANIA database and the co-expression network was further identified.

### STRING (https://string-db.org/)

In String database, the protein network of CDKs were analyzed, and the screening condition was a correlation greater than 0. 9. Co-expression analysis was also simultaneously to map co-expression, and integrate the differential expression of CDKs and their potential interactions (15).

### TIMER (http://www.timer.cistrome.org/)

TIMER 2.0 is an online resource database for analyzing immune cell infiltration in various cancers, applying deconvolution methods as a statistical method to infer the infiltration abundance of immune cells in tumor tissues from gene expression profiling (16). In this study, TIMER was used to evaluate the correlation of CDKs and infiltration of different immune cells in 458 CRC patients.

### Statistical Analysis

SPSS 22.0 statistical software was used for data analysis. The measurement data with mean and standard deviation (x± s), paired t test was used for comparison and c^2^ test or Fisher exact test for count data. Log-rank test for survival analysis examine and P<0.05 was defined as statistical significance. The correlation was evaluated with spearman rank correlation analysis. Deconvolution methods is adopted for infiltration abundance analyses of immune cells.

## Results

### Differential Expression of CDKs in Colorectal Cancer Patients

**Figure 1:**
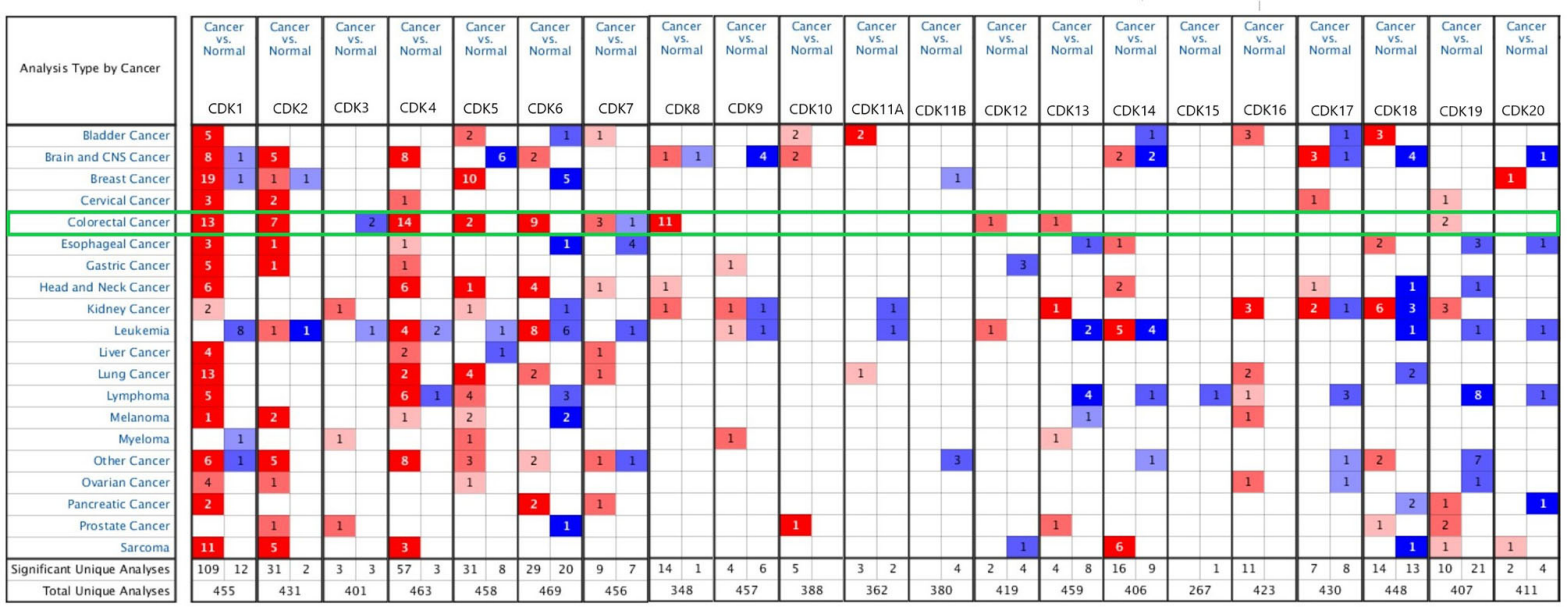
Expression of CDKs family members in different cancer types. The graphic shows the numbers of data sets with statistical changes in CDKs: Up-regulated expression of CDKs is red and down-regulated expression of CDKs is blue. p-value was set to 0.01, fold change was 2, and gene rank was10%, data type chose mRNA, and analysis type chose cancer vs. normal tissue. The green frame shows the analysis of CRC, mRNA levels of CDK1/2/4/5/6/7/8/12/13/19 were signifificantly increased in CRC tissues compared to normal tissues, and that of CDK3 were reduced in the former than in the latter.

The Oncomine database was used to screen for CDKs mRNAs differentially expressed between CRC and corresponding normal tissues (**Figure 1**). Compared to normal tissues, CDKs 1/2/4/5/6/8/12/13/19 were overexpressed, whereas CDK3 was downregulated in CRC tissues. Oddly, CDK7 was highly expressed in some patient samples and downregulated in others. The exact fold-changes and *P* values for differential CDKs expression are listed in **Supplementary Table 1**.

**Figure 2:**
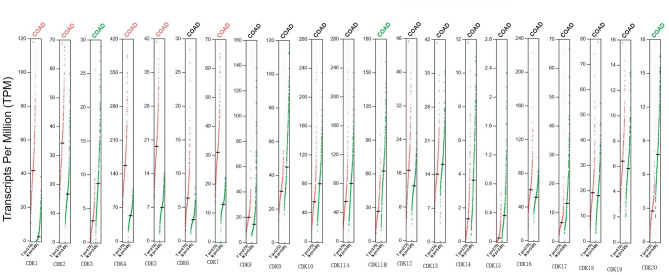
Expression of mRNA levels for CDKs in CRC patients (GEPIA). Scatter diagram showed that the mRNA expression levels of CDK1/2/4/5/7 were higher in CRC tissues than in normal tissues, and CDK3/11B/20 were lower in CRC tissues than in normal, upregulated (red) and downregulated (blue) (p < 0.05).

The GEPIA database was also employed to identify CDKs mRNAs differentially expressed between CRC and normal tissues. In partial accord with Oncomine results, CDKs 1/2/4/5/7 were overexpressed while CDKs 11B/3/20 were downregulated in tumor tissues (**Figure 2**).

**Figure 3:**
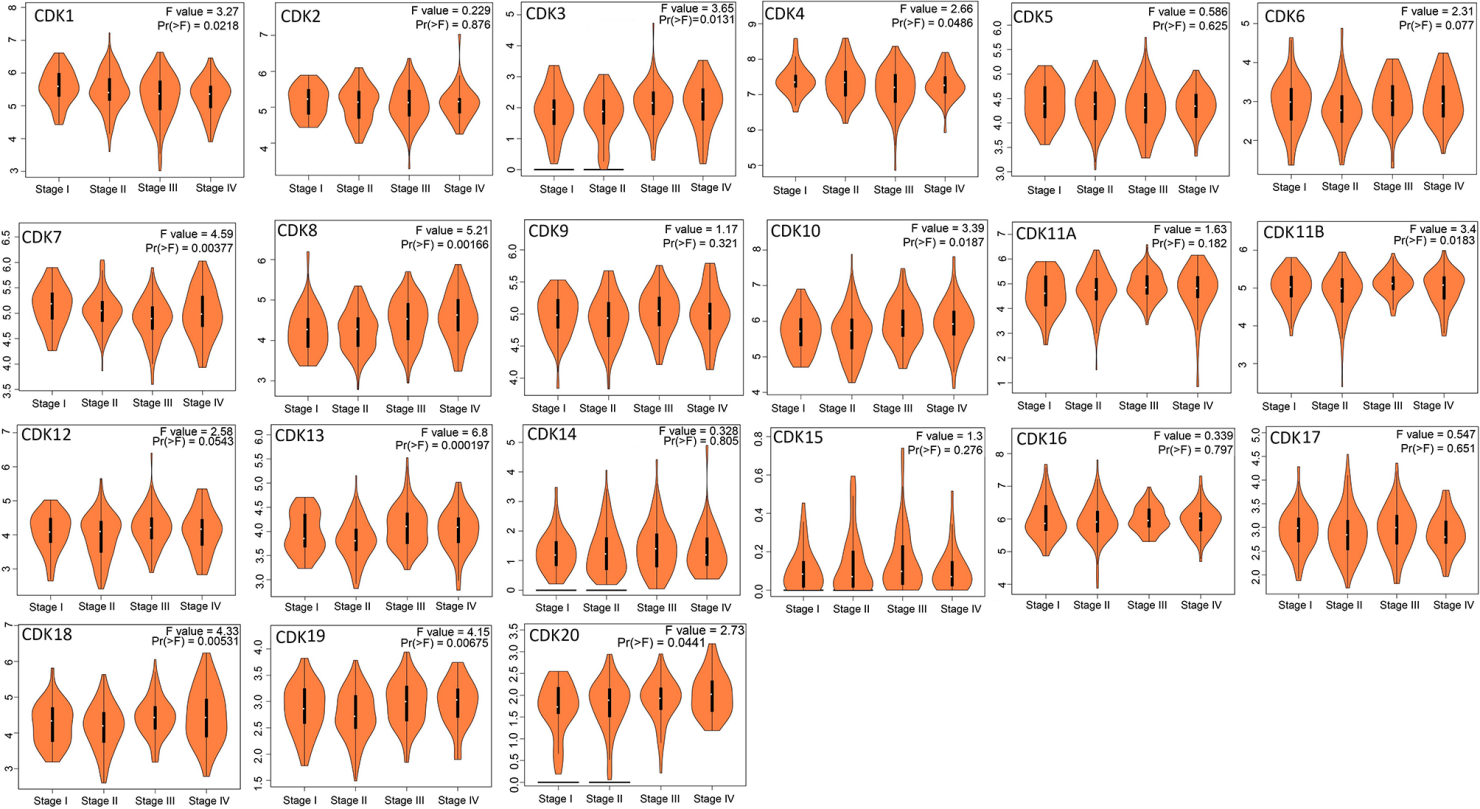
Relationship between CDKs expression and tumor stage of CRC patients (GEPIA). The mRNA levels of CDK1/3/4/7/8/10/11B/13/18/19/20 were related with the development of CRC (p < 0.05).

Correlation analyses revealed that expression levels of CDK7/8/18/19 were associated with clinical stage (**Figure 3**), suggesting that these CDKs may be useful prognostic biomarkers and potential therapeutic targets.

### Clinical Significance of CDKs mRNA Expression Profiles in Colorectal Cancer

**Figure 4:**
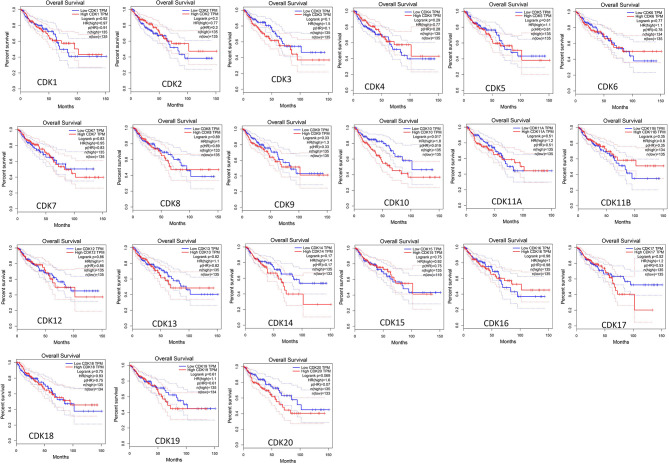
Correlations between the overall survival and mRNA expression of CDKs family members in CRC (GEPIA). High mRNA levels of CDK10 were signifificantly related to short OS in CRC patients.

The GEPIA database was adopted to evaluate correlations between differential expression levels of CDKs mRNAs and clinical prognosis. Patients expressing high level of CDK10 mRNA demonstrated significantly shorter overall survival (OS) (*P*=0.019), while other CDKs exerted no significant effect on OS (**Figure 4**) or disease free survival (DFS) (**Supplementary Figure 1**).

**Figure 5:**
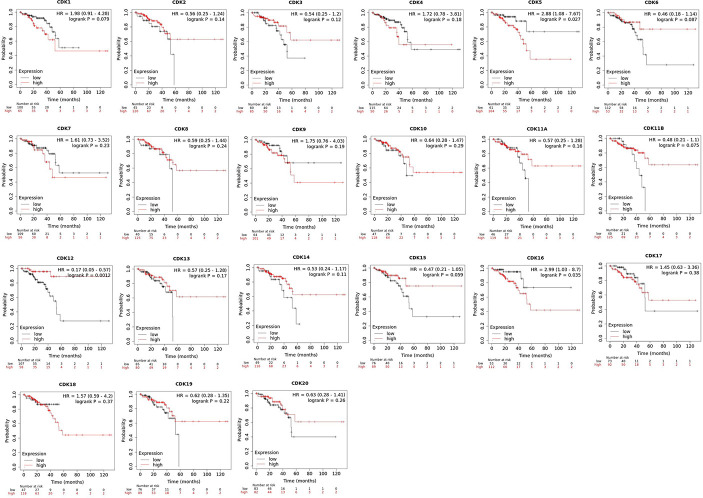
Role of CDKs in rectal cancer and prognostic evaluation (Kaplan-Meier plotter). High CDK5/16 and low CDK12 mRNA expression were signifificantly correlated with short OS in rectal cancer patients.

Furthermore, Kaplan-Meier plotter was also employed to analyze the correlations between CDKs transcription levels and clinical prognosis in rectum adenocarcinoma patients, a subgroup analysis. Rectal cancer patients with high expression levels of CDK 5 (p=0.027)/16 (p=0.035)exhibited shorter OS. However, high expression levels of CDK12(p=0.0012) exhibited longer OS (**Figure 5**).

### CDKs Gene Mutations and Protein–Protein Interaction in Colorectal Cancer Patients

**Figure 6:**
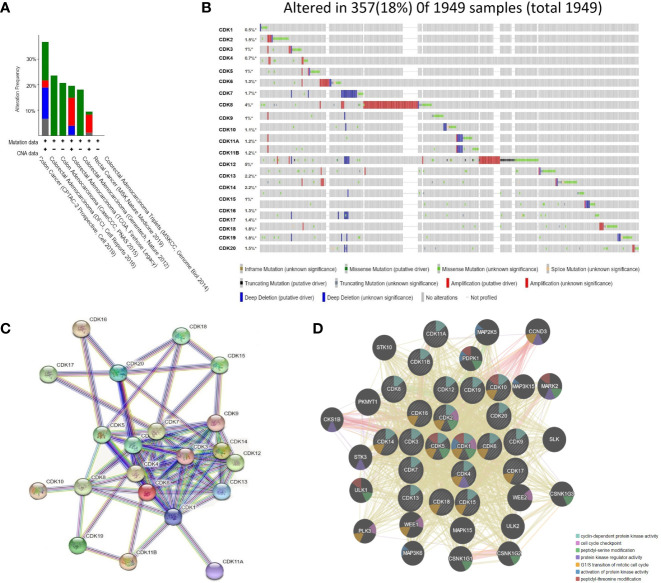
Analysis of CDKs gene mutation and Protein-Protein Interaction networks in CRC patients (cBioPortal and STRING). **(A)** Mutation analysis for CDKs in CRC subgroups. **(B)** CDKs were altered in 357 samples of 1,949 CRC patients, up to 18%. **(C)** Protein–protein interaction networks of different expressed CDKs. **(D)**. Protein-Protein interaction networks functional enrichment analysis of CDKs. * refered to containing unprofiled samples.

On-line analysis of CDKs gene mutations in CRC patients using the cBioPortal database revealed that individual cancer subtypes could present with multiple mutation patterns, and both CDKs gene deletions and mutations were found in colon and colorectal adenocarcinoma patients (**Figure 6A**). Among 1949 patient samples in the database, CDK gene mutations were found in 357 (18%), with the most common being CDK8 mutations (4%) and CDK12 (5%) (**Figure 6B**).

Protein-protein interaction (PPI) network analysis was conducted using the STRING website. Twenty-one nodes and 79 edges were obtained in the CDKs PPI network (**Figure 6C**). Co-expression analysis showed that multiple CDKs, and in addition to the relevant cell cycle-related function, CDKs family members were involved in amino acid polypeptide modification as well as the regulation of protein kinase activity (**Figure 6D**).

### Influence of CDKs on Immune Infiltration

**Figure 7:**
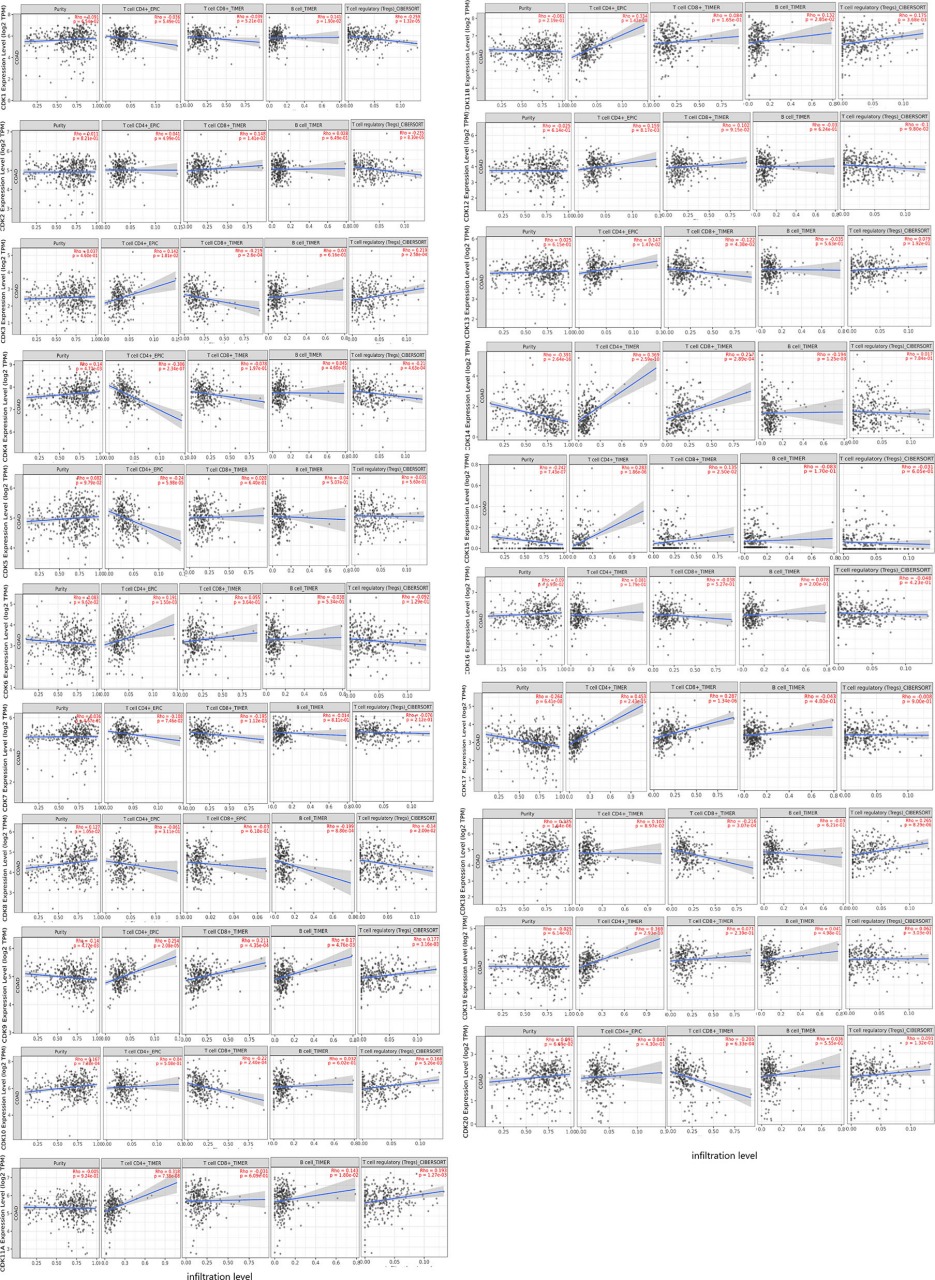
Correlations between the expression levels of CDKs and the abundance of immune cell infifiltration in CRC patents.

Tumor-infiltrating lymphocytes play an important role in the tumorigenesis and progression. The influence of CDKs expression profile on tumor immune cell infiltration was examined using the TIMER database (**Figure 7**). With the exception of CDK16, expression levels of all other CDKs were correlated with infiltration of various immune cells. Expression of CDK9 was positively correlated with infiltration of CD4+T cells (*P*=2.08e-05), CD8+T cells (*P=*4.35e-04), B cells (*P=*4.76e-03), and Regulatory T cells (Tregs) (*P*=3.16e-03), while expression levels of CDK11B (*P=*1.01e-07) and CDK15 (*P*=1.86e-06) were positively correlated with CD4+T cell infiltration. Expression of CDK14 was positively correlated with infiltration of CD4+T cells (*P*=2.59e-10) and CD8+T cells (*P*=2.89e-04), whereas the expression levels of other CDKs family members were negatively correlated with infiltration of one or more immune cell types. So to summarize, CDK9/14/17 were positively associated with the infifiltration of CD8+ T cells, while CDK3/7/10/13/18/20 were on the contrary. There are many family members of the CDKs associated with high infiltration of CD4 + T cells: CDK3/6/9/11A/11B/12/13/14/15/17/19, however, only CDK4/5 associated with its low infiltration. With regard to Tregs, CDK1/4/8 was associated with its low infiltration, while CD3/9/10/11A/11B/18 were associated with its high infiltration. Additionally, the infifiltration of B cells have a positive correlation with CDK1/9/11A/11B, but negatively correlation with CDK8/14.

## Discussion

Colorectal cancer is one of the most frequent malignant tumors in clinical practice, and the morbidity and mortality rates rank among the top 5 cancers worldwide (17). The diagnostic rate of colorectal cancer is low and the metastasis rate is high due to insignificant early clinical symptoms and lack of effective diagnostic biomarkers (18), contributing to the high mortality rate. Therefore, it is of great significance to identify novel specific biomarkers and intervention targets for diagnosis, prognosis, and treatment.

Cyclin-dependent kinases are serine/threonine protein kinases that participate in multiple biological functions, including cell cycle regulation and RNA transcription and processing. Under the action of various genetic and external stimuli, the regulatory mechanism governing CDKs expression may be disrupted, leading to uncontrolled cell division and proliferation (19–21) Twenty CDKs have been identified in human cells. Among them, CDK 1–6 and CDK 14–18 are directly involved in cell cycle regulation and cell division, whereas CDK 7–13 and CDK 19–20 mainly regulate gene transcription (22). Previous studies have demonstrated that the expression levels of CDK1/4 are upregulated in poorly-differentiated colorectal cancer cells (23–25). CDK5 participates in tumorigenesis *via* the ERK5-AP-1 signaling pathway (26, 27), Inhibitors of CDK5 have proven effective for treating colorectal cancer (28). These clinical results are consistent with our findings that CDK1/4 are highly expressed in colorectal tumor tissues and correlated with pathological stage, while CDK5 overexpression is associated with shorter OS.

Cyclin-dependent kinase 3 is also overexpressed in various tumor tissues, such as breast cancer and nasopharyngeal carcinoma (29, 30). However, the reported findings on CDK3 in cancer are inconsistent (31, 32). For instance, overexpression of CDK3 can suppress the migration and invasion of breast cancer cells. Our analysis indicated that CDK3 is actually downregulated in colorectal cancer tissues, which could enhance migration and invasive capacities. CDK8 regulates transcriptional process *via* associating with the mediator complex or phosphorylating transcription factors (TF). Overexpression of CDK8 has been observed in various cancers (33). CDKI-73, one of the most potent and pharmacologically superior CDK9 inhibitors, has demonstrated excellent anti-tumour efficacy against several types of cancers (34). In this study, therapeutic potential of CDK9 against CRC was evaluated. The results showed that CDK9 were closely related to lymphocyte infiltration in tumor tissue, such as CD4 + T cells, CD8 + T cells, et, but the expression level of CDK9 was neither increased nor decreased in the CRC. Cyclin-dependent kinase 12 involved in the regulation of the repair of homologous recombination genesis, by Inhibition of intronic polyadenylation. CDK12 plays different roles in tumorigenesis. Patients with wild-type rather than mutant CDK12 tumors were more sensitive to treatment (35, 36). In the present study, CDK12 overexpression was associated with higher survival rate, while patients with downregulated expression of CDK10 and 16 exhibited lower OS, suggesting that CDK10/16 and CDK12 can be used as prognostic biomarkers for colorectal cancer patients. Recent findings have shown that the expression level of CDK15 elevated in CRC, and there was a negatively correlation between CDK15 and overall survival. Further studies of the mechanism suggested that CDK 15 facilitated CRC tumorigenesis by phosphorylating PAK4 at the S291 residue (37). However, which is not consistent with the information analysis based on the existing databases, So more studies are needed to verify this result.

As an important part of the tumor microenvironment (TME), tumor-infiltrating lymphocytes (TIL) can both promote and hinder tumor progression as well as the therapeutic efficacy of anti-cancer treatments. There is a lot of research showing that T cell infifiltrate is important to therapy response, especially with high infiltration of CD8+ T cells was considered to be better prognostic factors in CRC patients (38–40). In the present study, CDK9/14/17 were positively associated with the infiltration of CD8+ T cells, which indicated that the high expression of CDK9/14/17 could favor immunotherapy and prognosis in CRC patients. In addition to T cells, other lymphocytes, such as B cells, and Tregs in tumor tissues, also play important roles in tumor treatment. The researches on the effect of tumor-infifiltrating B cells (TIL-B) in tumors are not consistent. The result of animal experment indicated B cells can inhibit anti-tumor immunity. however, high infiltration levels of TIL-B predict a favorable prognosis in clinical research (41, 42). Tregs, playing a negative regulatory role in immune response, is the main factor to maintain immunotolerance. Studies have shown that high infiltration by Tregs indicted poor survival in many tumors (43). CDK1/4/8 was associated with Tregs low infiltration, indicated that the high expression of CDK1/4/8 could improve the health-related quality of life, and prolonging the life of CRC patients.

## Conclusions

CDK 1 and 4 were highly expressed in colorectal cancer tissues and correlated with pathological stage, could be used as diagnostic biomarkers for CRC. Colorectal cancer patients with high expression levels of CDK 5/10/16 achieved lower OS, while high expression of CDK12 increased OS, hinting that CDK 5/10/12/16 can be utilized as prognostic biomarkers. With the exception of CDK16, other CDKs probably participate in and regulate immune cell infiltration into the tumor microenvironment. The findings in this study provide novel diagnostic and therapeutic targets for colorectal cancer.

## Data Availability Statement

The original contributions presented in the study are included in the article/**Supplementary Material**. Further inquiries can be directed to the corresponding author.

## Author Contributions

SL and LG conceived the idea. LG and YT designed the experiment. GL and ZQ collected data. LG analyzed the data, LG prepared **Figures 1**–**5**, GL prepared **Figures 6**, **7**. LG was a major contributor in writing the manuscript; YT contributed to discussion of the paper. All authors read and approved the final manuscript. All authors contributed to the article and approved the submitted version.

## Funding

This work was supported by Key Science and Technology Program of Henan Province (grant numbers 222102310098).

## Conflict of Interest

The authors declare that the research was conducted in the absence of any commercial or financial relationships that could be construed as a potential conflict of interest.

## Publisher’s Note

All claims expressed in this article are solely those of the authors and do not necessarily represent those of their affiliated organizations, or those of the publisher, the editors and the reviewers. Any product that may be evaluated in this article, or claim that may be made by its manufacturer, is not guaranteed or endorsed by the publisher.
