# Supplementary material for: Comprehensive Analysis of Role of Cyclin-Dependent Kinases Family Members in Colorectal Cancer
Source: Front Oncol. 2022 Jun 22;12:921710. doi: 10.3389/fonc.2022.921710 (PMC9258493; doi:10.3389/fonc.2022.921710)
Supplement: Supplementary file 1 [file DataSheet_1.doc]

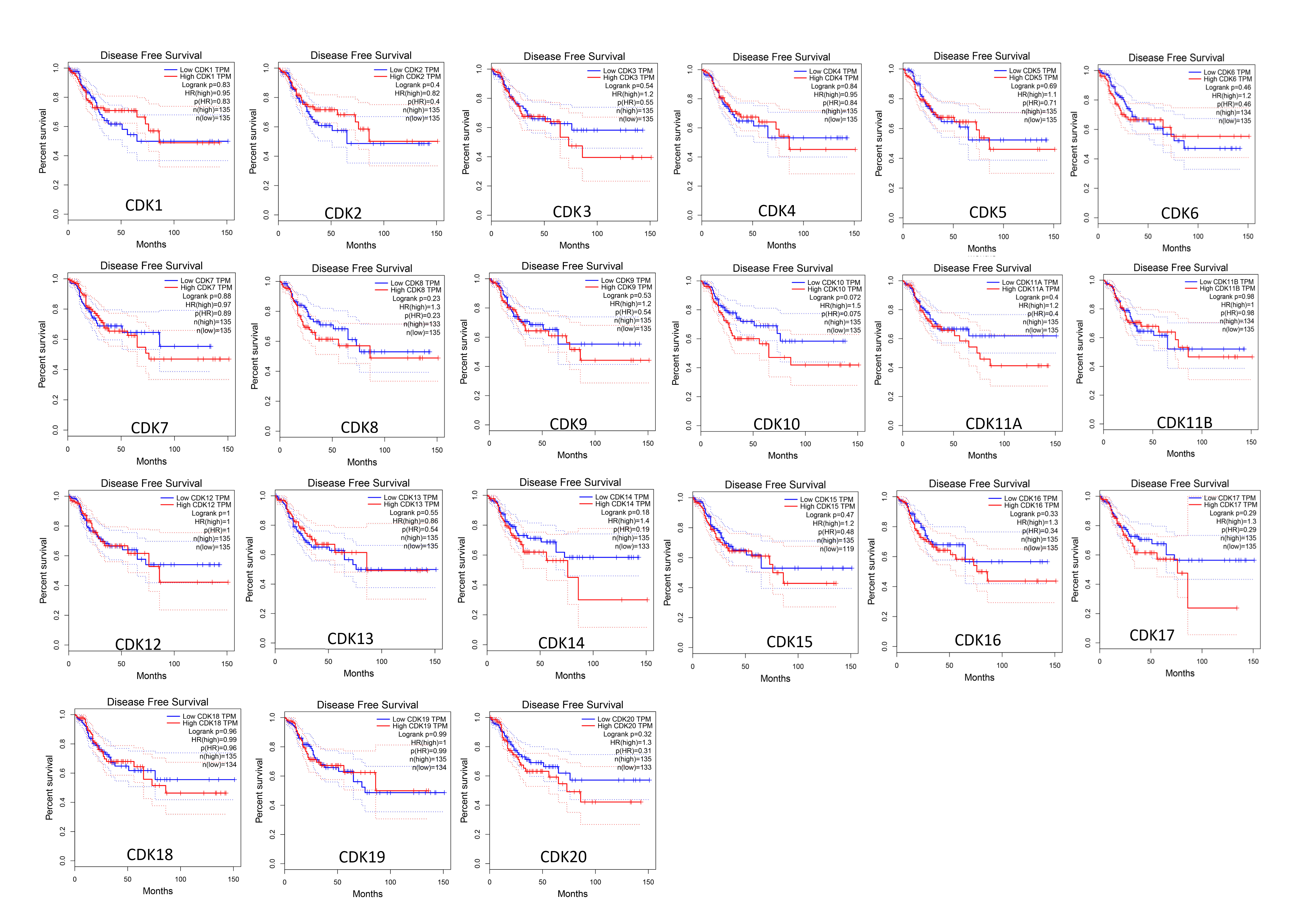


**Supplemental Figure 1.Correlations between the disease free survival and mRNA expression of CDKs family members in CRC (GEPIA).**

**Supplementary table1.The exact fold-changes and *P* values for differential CDKs expression in CRC.**

| Name | Types of CRC VS.Nomal tissues | Fold Change | P Value | T-test | Reference or Source |
| --- | --- | --- | --- | --- | --- |
|  | Colon Adenocarcinoma | 2.274 | 6.34E-13 | 10.804 | TCGA |
| CDK1 | Conlon Mucinous Adenocarcinoma | 2.695 | 7.81E-10 | 7.695 | TCGA |
|  | Colorectal Carcinoma | 4.476 | 6.020E-19 | 4.476 | Sabates-Bellver Colon |
|  | Rectal Adenocarcinoma | 2.897 | 1.96E-12 | 11.100 | TCGA |
|  | Cecum Adenocarcinoma | 2.148 | 4.50E-8 | 2.148 | TCGA |
| CDK2 | Colon Adenoma | 3.558 | 1.35E-9 | 16.606 | Skrzypczak |
|  | Conlon Carcinoma | 2.793 | 7.80E-9 | 12.773 | Skrzypczak |
| CDK3 | Colon Mucinous Adenocarcinoma | -1.118 | 0.872 | -1.152 | TCGA |
| CDK4 | Colon Adenocarcinoma | 3.374 | 3.27E-18 | 18.022 | TCGA |
|  | Colon Mucinous Adenocarcinoma | 3.249 | 8.81E-14 | 10.684 | TCGA |
| CDK5 | Colon Adenocarcinoma | 1.817 | 1.71E-17 | 12.928 | TCGA |
|  | Rectal Mucinous Adenocarcinoma | 4.934 | 6.42E-4 | 1.506 | TCGA |
| CDK6 | Colon Adenocarcinoma | 1.198 | 0.002 | 3.093 | TCGA |
|  | Cecum Adenocarcinoma | 1.183 | 0.051 | 1.688 | TCGA |
| CDK7 | Colon Adenocarcinoma | 1.368 | 8.47E-9 | 6.707 | TCGA |
|  | Rectal Adenocarcinoma | 1.436 | 5.50E-10 | 7.353 | TCGA |
| CDK8 | Colon Adenocarcinoma | 2.784 | 1.74E-6 | 15.785 | TCGA |
|  | Rectal Mucinous Adenocarcinoma | 2.323 | 3.26E-5 | 7.147 | TCGA |
| CDK9 | Colon Mucinous Adenocarcinoma | 1.440 | 1.05E-6 | 5.538 | TCGA |
|  | Rectal Mucinous Adenocarcinoma | 1.262 | 0.071 | 1.696 | TCGA |
| CDK10 | Colon Adenocarcinoma | 1.239 | 9.88E-5 | 4.066 | TCGA |
|  | Rectal Adenocarcinoma | 1.259 | 5.52E-4 | 3.432 | TCGA |
| CDK11A | Colon Adenocarcinoma | -1.179 | 0.998 | -3.125 | TCGA |
|  | Rectal Mucinous Adenocarcinoma | -1.015 | 0.548 | -0.125 | TCGA |
| CDK11B | Rectal Mucinous Adenocarcinoma | 1.737 | 0.003 | 3.153 | TCGA |
|  | Colon Adenocarcinoma | -1.116 | 0.793 | -0.830 | TCGA |
| CDK12 | Colon Adenocarcinoma | 1.630 | 2.77E-14 | 8.927 | TCGA |
|  | Rectal Adenocarcinoma | 1.801 | 1.46E-11 | 7.733 | TCGA |
| CDK13 | Colon Adenocarcinoma | 1.352 | 1.53E-6 | 5.441 | TCGA |
|  | Rectosigmoid Adenocarcinoma | 1.959 | 1.60E-10 | 12.451 | TCGA |
| CDK14 | Colon Adenocarcinoma | -1.754 | 1 | -6.022 | TCGA |
|  | Rectal Adenocarcinoma | -2.043 | 1 | -6.281 | TCGA |
| CDK15 | Colon Adenocarcinoma | -1.052 | 0.745 | -0.668 | TCGA |
|  | Rectal Mucinous Adenocarcinoma | 1.306 | 0.384 | 0.303 | TCGA |
| CDK16 | Rectosigmoid Adenocarcinoma | 1.600 | 0.048 | 2.663 | TCGA |
|  | Cecum Adenocarcinoma | 1.155 | 0.034 | 1.870 | TCGA |
| CDK17 | Colon Adenocarcinoma | 1.170 | 0.005 | 2.686 | TCGA |
|  | Rectal Adenocarcinoma | 1.132 | 0.024 | 2.039 | TCGA |
| CDK18 | Colon Adenocarcinoma | 1.094 | 0.091 | 1.360 | TCGA |
|  | Rectal Adenocarcinoma | 1.138 | 0.032 | 1.904 | TCGA |
| CDK19 | Colon Adenocarcinoma | 1.406 | 1.51E-5 | 4.556 | TCGA |
|  | Rectal Adenocarcinoma | 1.537 | 1.17E-6 | 5.593 | TCGA |
| CDK20 | Rectal Mucinous Adenocarcinoma | -1.385 | 0.997 | -3.354 | TCGA |
|  | Colon Adenocarcinoma | -1.735 | 1 | -7.170 | TCGA |
